# The Disease with a Thousand Faces and the Human Microbiome—A Physiopathogenic Intercorrelation in Pediatric Practice

**DOI:** 10.3390/nu15153359

**Published:** 2023-07-28

**Authors:** Vasile Valeriu Lupu, Lacramioara Ionela Butnariu, Silvia Fotea, Ionela Daniela Morariu, Minerva Codruta Badescu, Iuliana Magdalena Starcea, Delia Lidia Salaru, Alina Popp, Felicia Dragan, Ancuta Lupu, Adriana Mocanu, Tatiana Chisnoiu, Alexandru Cosmin Pantazi, Elena Jechel

**Affiliations:** 1 Pediatrics Department, Faculty of Medicine, “Grigore T. Popa” University of Medicine and Pharmacy, 700115 Iasi, Romania; 2 Faculty of Medicine, “Grigore T. Popa” University of Medicine and Pharmacy, 700115 Iasi, Romania; 3 Clinical Medical Department, Faculty of Medicine and Pharmacy, “Dunarea de Jos” University of Galati, 800008 Galati, Romania; 4 Faculty of Pharmacy, “Grigore T. Popa” University of Medicine and Pharmacy, 700115 Iasi, Romania; 5 Pediatrics Department, Faculty of Medicine, “Carol Davila” University of Medicine and Pharmacy, 020021 Bucharest, Romania; 6 Faculty of Medicine and Pharmacy, University of Oradea, 410087 Oradea, Romania; 7 Pediatrics Department, Faculty of Medicine, Ovidius University, 900470 Constanta, Romania

**Keywords:** systemic lupus erythematosus, microbiome, children

## Abstract

Numerous interrelationships are known in the literature that have the final effect of unmasking or influencing various pathologies. Among these, the present article aims to discuss the connection between systemic lupus erythematosus (SLE) and the human microbiome. The main purpose of this work is to popularize information about the impact of dysbiosis on the pathogenesis and evolutionary course of pediatric patients with SLE. Added to this is the interest in knowledge and awareness of adjunctive therapeutic means that has the ultimate goal of increasing the quality of life. The means by which this can be achieved can be briefly divided into prophylactic or curative, depending on the phase of the condition in which the patient is. We thus reiterate the importance of the clinician acquiring an overview of SLE and the human microbiome, doubled by in-depth knowledge of the physio-pathogenic interactions between the two (in part achieved through the much-studied gut-target organ axes—brain, heart, lung, skin), with the target objective being that of obtaining individualized, multimodal and efficient management for each individual patient.

## 1. Introduction

Being defined as a multisystemic inflammatory disease, juvenile systemic lupus erythematosus (SLE) presents a peak incidence during puberty (12.6 years) and an increased activity compared to the adult form. The evaluation of the activity of the disease is mainly done by measuring the specific antibodies represented by anti-nuclear (ANA) and, respectively, anti-double-stranded DNA (anti-ds-DNA) antibodies. Therefore, pediatric SLE (pSLE) needs a more aggressive therapeutic with the aim of preventing or limiting damage. These characteristics proved to be much more pronounced in the age groups under 5–7 years, where the low frequency of ANA stands out, which is doubled by the low titer of anti-ds-DNA and an increased rate of neuropsychiatric symptoms in contrast to renal and musculoskeletal damage. Regarding the determining factors of the condition, single genetic mutations (identified in >7% of the subjective), but also the combination of genetic predisposition and disturbing environmental factors (“aggressors”), can be incriminated in the development of SLE [1,2]. Affection occurs predominantly among prepubertal children and adolescents. In these age groups, the psycho-social impact is important and results from both the pathology and the treatment itself, as well as the resulting feeling of social isolation as a consequence of the different lifestyle. Thus, aware of the possible long-term repercussions, we consider it appropriate to emphasize the need to develop optimal scales for evaluating and improving the quality of life [3]. Pharmacotherapy based, similarly to adults, on steroids and immunomodulators, with doses adapted according to age and comorbidities, improved survival at 10 years, being estimated at 90% in this age group [4]. Besides this, the specialized literature currently places the influence played by the modulation of the microbiome (with the help of prebiotics, probiotics, symbiotics or fecal microbiota transplant from healthy people) in the clinical-biological evolution of SLE. This benefit is partly explained from the perspective of finding dysbiosis among the triggering or disturbing factors of lupus.

The microbiome–host inter-relationship is in continuous evolution parallel to that of the human body since the intrauterine period and until the age of senescence, an aspect that validates the involvement of the former in various physio-pathological processes (immune system diseases, neurological and metabolic diseases and even diseases from the oncological sphere). The main ways in which the microbiota exerts effects on the body in all stages of life are represented by influencing the metabolic balance, modulating the synthesis and absorption of vitamins (by this means regulating functions such as coagulation) or imprinting the balance of T helper lymphocytes (1/2/17) and regulatory T cells. To these, the ability to influence intestinal maturation and the diversity of food digestion products such as short-chain fatty acids (butyrate, propionate, acetate) is added. The latter exert their functions on the integrity of the intestinal barrier (being an important source of energy), the inflammatory balance, as well as body weight. Unlike the components of the external environment that must cross certain barriers (epithelial/intestinal) to interact with the internal environment, the endogenous microbiota can facilitate homeostasis imbalances much more easily, dictated by an inversion of the ratio (“dysbiosis”) beneficial bacteria/ harmful bacteria [5]. Thus, the human microbiome is made up of bacterial species specific to each site in the body (skin, oral cavity, gastrointestinal tract, upper and lower respiratory or genitourinary tract), whose ratio varies depending on age, type of birth, early childhood and external environment [6]. Due to its complexity and variability, the human microbiome represents a central research pillar of the last decades; the evidence in this sense resides in the development of an integrative study project, with a duration of 10 years, carried out in two phases, which is focused on understanding dynamics and impacts on health (e.g., inflammatory bowel diseases, pre-diabetes) [7]. Taking into account the impact played by the two entities previously developed during childhood and later during adulthood, the present narrative review aims to bring the microbiome–SLE connection up to date by exposing the most recent works in the field of interest, with the aim of understanding it and how it can be influenced for prophylactic/curative purposes.

## 2. Materials and Methods

We performed a narrative review of the specialized literature using the PubMed, ScienceDirect and Oxford Academic databases to identify relevant articles related to how dysbiosis can influence the emergence and evolution of autoimmunity among pediatric patients with SLE. Searches focused on keywords and phrases frequently used to describe SLE, dysbiosis and the main directions to follow in their support and modulation (e.g., pediatric systemic lupus erythematosus, autoimmune disease, disease of a thousand girls, intestinal dysbiosis, oral dysbiosis, human microbiome, microbiome and autoimmunity, microbial sites, microbiome modulation, diet in SLE, probiotics/prebiotics/symbiotics in SLE, fecal microbiota transplantation, nutrient supplementation, modern therapies), as well as terms useful in directing to information about the pathogenesis of SLE and pathophysiological mechanisms behind the microbiome–immune system interaction (e.g., autoimmunity, predisposing factors, innate immunity, adaptive immunity, systemic inflammation, microbiome exploration, dysbiosis-associated pathologies, bacterial translocation, molecular mimicry). Thus, we brought together in the same review current topics intensively studied in medical research that include the human microbiome and ways of modulating it with the aim of maintaining the balance of harmful bacteria/beneficial bacteria. We have chosen to practically exemplify this presentation of information by making a correlation between dysbiosis and organic diseases, with an emphasis on the description of the implications in the potentiation and management of autoimmunity (respectively of SLE). Broadly speaking, the inclusion criteria concerned study groups made up of children (0–18 years); although, where clinical exploration was limited, we chose to include results obtained on adult groups or murine models, with the aim of covering the informational bias. The present work is therefore a crossroad of the current information regarding the pathogenesis, diagnosis and management of SLE described from the perspective of a less exploited causal relationship with dysbiosis, especially in pediatric practice.

## 3. Epidemiology

With a high mortality rate, both in comparison with the general population and with the adult form of the pathology, juvenile SLE represents approximately 15–20% of all lupus cases, with an incidence of 0.36–2.5 and a prevalence of 1.89–34.1 per 100,000 children [1]. Regarding the division by gender and ethnicity, less represented in early childhood compared to late childhood and adult life, the ratio is against girls (10 times higher prevalence than among boys) and Black/Asian ethnic groups compared to white Caucasians [2,4].

Regarding the complications of SLE, especially lupus nephritis, Hiraki LT. et al. objectify an increased proportion of positive cases in the research group (respectively, 37% of the total number of subjects), with a distribution dependent on the patient’s age, gender and demographic location. Thus, a 4.5 times higher prevalence is reported in girls compared to boys [8]. Similarly, the study of a cohort made up of children from New Zealand confirmed a two to four times higher incidence rate among them, namely Asians, compared to the European population, a burdened ratio and increased mortality due to the development of severe kidney lesions and lupus nephropathy [9].

The factors that increase the risk of developing complications are not yet fully defined, but it is unanimously accepted that they are part of the large family of genetic and environmental factors. This hypothesis complicates the work of research professionals because research in the field of the human microbiome has similarities with that of molecular epidemiology or the genome, but the results are much more variable in time and space (exogenous environment/various anatomical sites), individual architecture, component genes and diet-dependent drugs, such as antibiotics, infections or foreign substances, all of which lead to imprinting the health of the subject by influencing the metabolism [10,11,12].

## 4. Pathogenesis

The physio-pathological cascade encountered in SLE is a multivalent one, the homeostasis of the internal environment being disturbed on various levels, among which we note the impact of genetic factors such as mutations (protein kinase C delta-PRKCD, Ras, three prime repair exonuclease 1-TREX1, Fas cell surface death receptor-FAS, FAS-ligand, deoxyribonuclease 1), polymorphisms or aneuploidy that can determine family aggregates prone to certain diseases. While monogenic SLE (found in 7–8% of pediatric cases, compared to 1–4% of cases in adults) is caused by disruption of the genes involved in the complement pathway (C1q, C1r, C1s, C2, C4A and C4B), nucleic acid metabolism, apoptosis and immune tolerance reflected on the activity of B and T lymphocytes, the majority of SLE cases meet a coexistence of the involvement of genetics and additional factors in shaping the etiology. The genetic component can partly explain the division of the risk of the disease depending on sex, with current research emphasizing the pivotal role of the X chromosome in the pathogenesis, an aspect certified by the escalation of up to 14 times the cases of SLE in the male population diagnosed with Klinefelter syndrome compared to boys that have a normal karyotype (similar data being observed in girls with a supernumerary X chromosome, in contrast to those with Turner syndrome, where the reported prevalence is reduced). The genetic factors are doubled by the epigenetic component, the understanding of which is useful in order to elucidate the discrepancy between SLE incidents in homozygous twins, represented by DNA methylation, post-translational histone modification and the presence of non-coding micro-RNA sequences [1,13,14,15].

**Figure 1:**
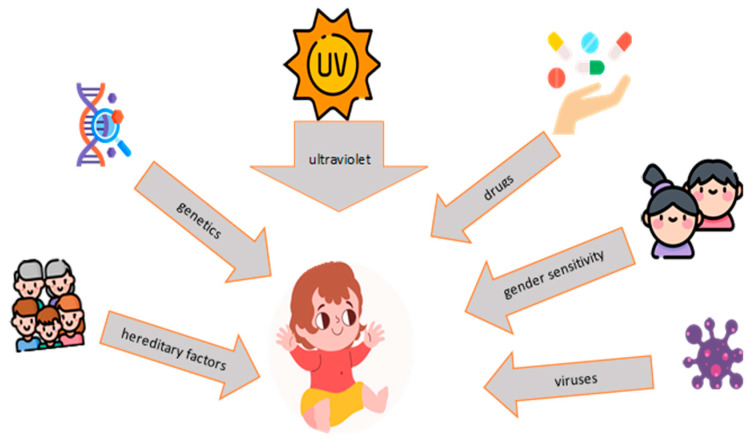
Predisposing factors in the pathogenesis of SLE.

In the pathogenesis of SLE, both innate and adaptive immunity are disrupted, resulting in the production of antigen–antibody complexes, activation of dendritic cells, abnormalities of phagocytes, overexpression of interferon (IFN) type 1, cytokines such as interleukin (IL)-1, IL -2, IL-4, IL-6, IL-8, IL-10, IL-17, IL-18, IL-21, IL-23 and activation of T-helper cells [16,17]. The epigenetic component can be disturbed by numerous exogenous factors (ultraviolet radiation, stress, smoking, infections, drugs), among which we emphasize the intestinal microbiota [18] (Figure 1). The variability of the two components (the host’s immune system and the microbiota) is in a perpetual change dictated by the food constituents, the health status of the individual and, respectively, the genetic predisposition. Furthermore, the two can act synergistically in order to modulate SLE activity (assessed by the dose of autoantibodies) among confirmed patients or their first-degree relatives. In this sense, although diet and genetic predisposition can influence the risk of SLE, Ogunrinde E. et al. underline the existence of microbial flora disturbances among first-degree relatives, not diagnosed with SLE (parents/children), of patients with a confirmed diagnosis. These disturbances are mainly attributed to the plasma translocation of microbial components, thus identifying differences and an increase in the plasma levels of autoantibodies in agreement with the increase in the levels of lipopolysaccharides. The hypothesis that resides in this correlation is the possible ability of the components in circulation to activate the system. A discrepancy was also observed between the microbial diversity and the level of autoantibodies in the three analyzed groups (healthy, first-degree relatives and patients with SLE), with the difference between the latter being partly attributed to the consequences of pharmacological therapy. In agreement with them, Clancy RM. et al. underline the importance of mimicry (cross-reaction to microbial peptides from the oral or intestinal flora) in defining the microbiome–autoimmunity relationship, which seems to modulate the transition from latent disease to severe clinical damage among mothers who give birth to children with neonatal lupus [19,20,21,22].

## 5. Diagnosis

In this study, the diagnostic lines follow two entities concurrently, namely SLE and systemic microbiota confined to various sites in the body. For an easier understanding, we will present in the following (Table 1) the main clinical aspects, investigations and diagnostic criteria that must be carried out/fulfilled in order to accurately define the notion of juvenile systemic lupus erythematosus and dysbiosis.

## 6. The Role of the Microbiome

The human microbiota, as we stated previously, is divided according to the sites of interest, the disruption of its homeostasis mainly in response to changes in environmental factors, diet or antibiotic therapy, with the effect of increasing the risk of atopy, autoimmunity, heart diseases or malignancy [26,27]. Ever since the intrauterine period, this living microenvironment undergoes development and adaptation processes that lead to its maturation over time, with the establishment of a balance between commensal and pathogenic organisms that are vital in the physio-pathogenesis of multiple diseases (Table 2).

Taking the intestinal microbiota (in a perpetual change until the age of 3 years, later with small variations) whose relationship with the maternal component is intensively studied as a model, Kim H. et al. have brought to attention, in addition to the impact played by living in the vicinity of pets, the influence of the prenatal and perinatal period in shaping the predisposition towards certain pathologies. In this sense, in addition to its role in nutrition and the maternal–fetal emotional bond, breast milk (colonized with *Streptococcus*, *Staphylococcus*, *Propionibacteria*, lactic bacteria and *Bifidobacterium*) proved to be an important prebiotic, probiotic and vector of vertical bacterial transmission, modulating *Bifidobacterium*/*Firmicutes* balance, with the cessation of breastfeeding leading to the maturation of the intestinal microbiota with the inversion of the ratio of the two, in detriment of *Bifidobacterium*. Antepartum, intrapartum or postnatal antibiotic therapy, as well as antifungal therapy, also lead to the reduction of *Bifidobacterium*, *Bacteroides* and *Actinobacteria* species, promoting the dominance of *Enterococcus*, *Clostridium*, *Proteobacteria* and *Firmicutes*. A benefit of natural food is the objective of a reduced rate of colonization with *Escherichia coli*, *Clostridium difficile*, *Bacteroides* and lactobacilli. Last but not least, the type of birth and the gestational age seem to influence dysbiosis in the same way as previously described, being thus preferable to natural birth that promotes fetal insemination with vaginal bacteria, to the detriment of cesarean surgery, considered a trigger factor for imbalance [28,29,30,31,32]. After the threshold of 3 years and until adulthood, Derrien M. et al. note a gap in the collection of data regarding the structure of the microbiome, while at the same time reiterating its evolutionary directions [33].

### 6.1. Skin

The skin represents the system with the widest distribution in the body, modulating through its functions both the interaction between the exogenous and the endogenous environment, as well as the variability and viability of the microorganisms that can colonize it permanently or accidentally. This inter-relationship can be influenced by ultraviolet radiation, thermogenesis, humidity and local pH. The predominant bacterial species at its level are *Actinobacteria*, *Firmicutes*, *Proteobacteria* and *Bacteroides*, while among fungi we note *Malassezia*, *Cryptococcus*, *Rhodotorula*, *Aspergillus* and *Epicoccum*. Dysbiosis at this level intervenes in the pathogenesis of conditions such as atopic/seborrheic dermatitis, acute urticaria, alopecia, acne, psoriasis or skin malignancies [34,35,36,37]. Besides the well-known factors that modulate skin colonization, Bouslimani A. et al. also underline the impact of chemical products applied to the skin on biodiversity, the level of steroids and pheromones, with particular reference to facial care products and deodorants [38]. Regarding the effects of the establishment of optimal treatment, a randomized study regarding the restoration of balance in children with atopic dermatitis (moderate/severe) treated with emollients, anti-inflammatory agents (corticosteroids) and antiseptics (diluted bleach) shows that the levels of *Staphylococcus aureus* at the end of the follow-up period were lower in the case of patients who received antiseptics, unlike those who received standard treatment [39]. Taking note of these findings, we bring into consideration the current lines in restoring microbial homeostasis, namely probiotics, phage therapies, humanized monoclonal antibodies against bacterial toxins or quorum sensing inhibitors [40].

Regarding autoimmune diseases, Zhou HY. et al. note the presence of a particular skin microbiota with affected bacterial diversity both in eruptive and non-eruptive areas in the case of patients with SLE, unlike healthy controls and those with rosacea (chronic inflammation of the skin that predominantly affects the medio-facial area, being characterized by erythema, telangiectasias, eruptions of small pimples and papules and, in advanced cases, increase in the volume of the nose -rhinophyma-). From the point of view of the identified microorganisms, a discrepancy was objectified between the regions affected by SLE and the unaffected skin areas, which was characterized by the increase of the genus *Halomonas* together with the decrease of the genera *Pelagibacterium*, *Novosphingobium* and *Curvibacter* [41].

### 6.2. Respiratory System

Having a diminished immune defense during childhood, especially at the level of the mucous membranes, objectified by the increased incidence of various pathologies in the respiratory sphere, maintaining the balance of microorganisms that usually or occasionally colonize the respiratory tract (spread out between the nostrils and the pulmonary alveoli, with a role in humidification air, filtering inhaled particles and oxygen-carbon dioxide exchange) represents a topic of interest in current pediatric practice by proving its involvement both in its maturation and in the regulation of immunity. And at this level, the type of birth, nutrition and antibiotic therapy, in addition to seasonal influences, vaccination, history of respiratory tract infections, living together with siblings, frequenting collectives and exposure to smoke have been shown to have a dynamic imprint on the microbial balance (*Staphylococcus*, *Corynebacterium*, *Dolosigranulum*, *Moraxella*, *Haemophilus*, *Neisseria*) of the upper and lower respiratory tract, which was considered a sterile structure until recently [42,43]. Cao W. et al. underline that pathogenic microorganisms are mainly confined to the lymphoid organs (tonsils, adenoid vegetations, oropharynx or nostrils) [44]. Dysbiosis within this system can lead to diseases such as acute otitis media, chronic rhinosinusitis, bronchiolitis, pneumonia, asthma or post-injury residual sequelae (e.g., SARS-CoV-2 infection, the severity of which was inversely correlated with the diversity of the oropharyngeal microbiome) [42,43,45,46]. It is also worth mentioning the connection between SARS-CoV-2 and SLE, both the infection and the vaccination, seemed to influence the course of the patients despite the summary data from the literature, especially regarding the pediatric population [47].

Being the well-known connection between the intestine and the lungs, the current specialized literature recommends the maintenance of balance and the modulation of the former (with the help of probiotics based on *Lactobacillus* and *Bifidobacterium*) during the evolutionary course of children with recurrent respiratory diseases [48,49].

### 6.3. Genitourinary System

The genitourinary tract is made up of the urinary system (kidneys, ureters, bladder and urethra) in close connection with the genital organs (internal and external, different between the sexes) and the digestive tract, especially at the level of the external openings. Thus, the microbiota of the three systems, although individual for each one, can show similarities, potentiating each other. In the female sex, the main organs whose bacteriological study is easy are the vagina and the uterus, which have structures that show variable colonization through a retrograde mechanism, hematogenous transmission or the seminal fluid, which, after the onset of puberty, is dependent on the phases of the menstrual cycle and the presence/absence of possible pregnancies, and which are separated from each other by the cervical mucus plug that acts as a barrier to the ascent of pathogens (*Ureaplasma*). Microorganisms present at this level are *Lactobacillus*, *Bifidobacteriaceae*, *Gardnerella vaginalis*, *Actinobacteria*, *Prevotella*, *Enterobacter*, *Streptococcus*, *Proteobacteria* and *Bacteroides,* whose fragile balance is incriminated in the pathogenesis of bacterial vaginitis, pelvic inflammatory disease, hysteromyoma, endometriosis, adenomyosis, transmission infection sexual or those with the human papillomavirus, which can evolve into cervical dysplasia [50,51,52,53]. Also, vaginal dysbiosis can be incriminated in the production of neonatal infections, spontaneous abortions and premature birth, in the case of young mothers [54]. Ling Z. et al. record a more pronounced vaginal dysbiosis, in contrast to that found in the examination of fecal matter in the case of patients with SLE, characterized by a different predominance of frequently encountered species, but also by an intense association between the vaginal microbiome of the patients and the immunological picture of the disease (negative correlation between the C4 fraction of complement and *Bacteroides*, *Escherichia* and *Shigella*) [55]. In boys, the microbiota of the genital tract remains open to research, in part due to the invasive methods required to study it. As a compromised option, the seminal fluid was studied, with the results showing the presence of *Lactobacillus*, *Bifidobacterium*, *Faecalibacterium*, pathogenic microorganisms characteristic of sexually transmitted diseases, *Enterobacteriaceae*, *Escherichia coli*, *Ureoplasma*, *Prevotella*, *Corynebacterium*, *Enterococcus* and *Staphylococcus aureus*, microorganisms whose balance imprints the development of inflammatory conditions, male fertility and even the risk of malignancy in the prostate [56].

While the colonization of the urethra mostly respects the bacterial species identified at the level of the genital system, we emphasize here the need to know the variations of the bladder microbiome and urinary pH, depending on age and pathogen, respectively. A good diagnosis of these leads to the prophylaxis and optimal treatment of renal lithiasis or urinary tract infections, thus eliminating the risk of long-term complications such as the chronicity of the condition, renal scars or resistance to antibiotics [56,57,58,59].

### 6.4. Gastrointestinal Tract

Starting from the upper orifice, the microbial components that populate the oral cavity of children are in constant evolution, the key moments of which are birth (the intrauterine colonization model is still under research, with the similarity between the placental microbiome and the oral microbiome of maternal origin being certified), with the dental eruption and the finalization of the dentition, being, however, also influenced by diet, type of birth, environmental factors, ethnic or geographic belonging, genetic determinants and horizontal transmission from the people with whom they interact [54,60]. Thus, divided by age groups, among the most known microorganisms that colonize the oral cavity are bacteria (*Streptococcus*, *Staphylococcus*, *Fusobacterium*, *Veillonella*, *Haemophilus*, *Escherichia coli*, *Pseudomonas* and *Lactobacillus*), viruses (rotavirus, norovirus, hepatitis C virus, herpes simplex 1/2, flu, Coxsackie A or Epstein–Barr) and fungi (*Candida*, *Cladosporium*, *Saccharomycetales*, *Aspergillus* and *Cryptococcus*) [54].

The main diseases in which the microbiome of the oral cavity plays an essential role are certified to be dental caries that appeared during early childhood, inflammatory bowel diseases (Crohn’s disease, ulcerative colitis), post-infectious irritable bowel syndrome, celiac disease, diabetes, autism, Henoch–Schonlein purpura, Wiskott–Aldrich (defined as the presence of micro-thrombocytopenia, recurrent infections and eczema), appendicitis and sleep apnea syndrome. In their production, the main microorganisms incriminated are *Streptococcus (mutans*, *salivarius*, *sobrinus*, *parasanguinis)*, *Lactobacillus*, *Bacteroidetes*, *Vaillonella*, *Candida albicans*, *Prevotella*, *Limnohabitans*, *Rothia*, *Neisseria*, *Pasteurella stomatis*, *Spirochaets* or *Campylobacter*, doubled by a decrease in the abundance of *Actinomyces*, *Corynebacterium*, *Haemophilus*, *Eikenella*, *Ramlibacter*, *Mucilaginibacter*, *Proteobacteria*, *Pseudomonas*, *Moraxellaceae*, *Fusobacterium* and *Firmicutes*, which are currently intensively studied components, the variety of which proves to be different even in comparison with first-degree relatives and is the basis of the development of new biomarkers used in microbiota research, but also of the current principles of its modulation, through food means or substitution (probiotics, prebiotics, symbiotics or transplant of fecal matter) [54,61,62,63,64,65,66]. Knowing the characteristics of oral dysbiosis during the evolution of various pathologies, as well as the mechanisms of action through which it exerts its influence, has a vital role in the study and development of new therapeutic targets, the hypothesis exemplified by Xiao E. et al. on murine models, with reference to the diabetes-IL-17 activity-microbiota triad, where the de-escalation of the inflammatory and periodontal destruction processes was demonstrated together with the inhibition of IL-17 (cytokine with a role in promoting the inflammatory process and, indirectly, osteoclast activity) through means of monoclonal antibodies directed against them, effects doubled by a decrease in the pathogenesis of the oral microbial flora [67].

With reference to the stomach, the main lines of research regarding bacterial colonization include its modification in *Helicobacter pylori* (*H. pylori*) infection, but also in chronic gastritis, duodenal ulcer or carcinogenesis, conditions in which the microbiota has been shown to have an altered diversity compared to the batches of control subjects, with part of its components showing causal relationships with the incriminated pathologies (e.g., *Bacteroides—H. pylori* in children or *Methylobacterium* in gastric carcinogenesis, being also a negative prognostic marker) [68,69,70]. About the influence of *H. pylori* and gastric dysbiosis among duodenal ulcer patients, Zheng W. et al. postulated that in this situation, the character of infected/uninfected has the potential to modulate the community of genotoxic bacteria present at the level of the microbiota [71]. A role in this process seems to be played by iron, a constituent that, when found in low quantities, imprints the carcinogenic potential of *H. pylori*, possibly through the interaction with the metabolism of bile acids (especially deoxycholic acid) [72]. Due to the ever wider spread of *H. pylori*, doubled by the negative impact of the infection and the development of antibiotic resistance, it is necessary to know alternative therapies such as probiotics based on *Limosilactobacillus reuteri*, a Gram-positive bacterium resistant to gastric and bile juice, which maintains homeostasis in the environment by inhibiting the development of pathogenic species, while at the same time increasing adherence and therapeutic efficiency by improving digestive symptoms [73].

Dysbiosis at the intestinal level has been shown to be involved in multiple pathologies, starting from local ones (inflammatory intestinal diseases, celiac disease), osteoarticular (osteoarthritis), skin (psoriasis, acne vulgaris), vascular (atherosclerosis or thrombosis), chronic renal, hepatic, pulmonary (obstructive diseases, asthma) and metabolic (obesity, diabetes, dyslipidemia) and culminating with those in the neuropsychiatric sphere (depression, Alzheimer’s or Parkinson’s disease, autism, schizophrenia, multiple sclerosis) or neoplastic (oral, esophageal, pulmonary, pancreatic, colorectal), with all these processes being under the empire of the main axes formed by intestine and lung, brain, heart or skin [54,74,75,76]. From this wide range of diseases whose pathogenic process is based on disturbances in the intestinal microbiota caused by various individual or environmental factors, we note autoimmune diseases such as SLE, anti-phospholipid antibody syndrome, Sjogren’s syndrome, systemic sclerosis or rheumatoid arthritis, which appeared as an effect of the bidirectional microbiome–immune system relationship [77,78]. The connection between the components of the microbiome and SLE was also demonstrated with the help of a randomized study carried out by Xiang K. et al.; the authors underlined the possible existence of both provocative and protective factors that can guide the appearance and evolutionary course of the condition [79].

Taking the microbiome–SLE relationship as a model, a first change objectified by current studies is the inversion of the relationship *Firmicutes*/*Bacteroidetes,* overwhelmed by the escalation of species such as *Rhodococcus*, *Eggerthella*, *Klebsiella*, *Prevotella*, *Eubacterium* and *Flavonifractor* and the reduction *Lactobacillaceae*, which is a dysbiosis that leads to the potentiation of the chronic inflammatory response and the decrease of immune tolerance, with the increase of anti-double-stranded DNA antibodies and possible imprinting of renal function [77,80]. Chen BD. et al. also draw attention to the existence of pathogenic species such as *Clostridium* ATCC BAA-442, *Atopobium rimae*, *Shuttleworthia satelles*, *Actinomyces massiliensis*, *Bacteroides fragilis* and *Clostridium leptum* in samples collected from patients positive for SLE, compared to healthy subjects, with their levels decreasing after treatment [81]. The main mechanisms by which microbial metabolites (such as short-chain fatty acids, free fatty acids, amino acids and arachidonic acid) interfere with autoimmune processes mediated by T, B lymphocytes, dendritic cells or macrophages have been incriminated to be translocation, molecular mimicry and stimulation antibody production due to the presence of various epitopes. The biological arguments brought forward in favor of the incrimination of bacterial translocation consist of the objectification of increased levels of procalcitonin, a marker of inflammation and damage to the intestinal barrier, together with the escalation of CD14 and α1-acid glycoprotein values, although the subject is still being researched. Regarding the microbial metabolites involved in various pathologies, in SLE a vital role seems to be played by short-chain fatty acids (acetate, propionate, butyrate) and polyamines due to the effects exerted on autoimmune processes, promoting the integrity of the intestinal barrier, both being an important source of energy. Modulation of immune functionality is achieved by decreasing the production of pro-inflammatory cytokines (IL-6, IL-12, IL-17, IFN-γ and tumor necrosis factor α) in parallel with the promotion of anti-inflammatory cytokines (TGF-β and IL-10). In this sense, the hypothesis of the benefit obtained from the involvement of the two constituents in the diagnostic and therapeutic course of the condition is raised. At the same time, by studying a group of 61 pediatric patients, Wen M. et al. note a decrease in the values of essential amino acids (especially Valine, Leucine, Tryptophan and Phenylalanine, whose level is strongly correlated with immune, metabolic, neuronal activity and, in part, with favoring the presence and activity of certain pathogenic agents) in the plasma accompanied by the intensification of their presence in feces; together with the growth of Proteobacteria (genus *Sphingomonas*), which interferes with the digestion and absorption of proteins, the data regarding the model of change in diversity between positive subjects for SLE and healthy controls is contradictory to other studies with similar themes in the literature, which is possibly due to the influence of age and gender differences between the groups. Research in the gastrointestinal field associates the disturbance of the balance of amino acids and fatty acids with symptoms such as abdominal distension, pain, nausea, vomiting and anorexia, which were found in the clinical picture of SLE. The biological profile reveals an increased prevalence of lipid metabolism disturbances, with bile acids (deoxycholic, isohyodeoxycholic and arachidonic) being strongly correlated with the SLEDAI score, an aspect doubled by the discordance identified between the metabolites present in the serum and those detected in the feces [82,83,84,85,86,87,88,89,90].

Although it targeted a group of subjects over 18 years old, we consider it appropriate to discuss the results of the work done by Liu F. et al. [88] with reference to the microbial variation of the intestine and saliva between patients diagnosed with SLE and controls due to the strict inclusion characteristics in the study and the research directions launched, which are aspects that we consider still under research in pediatrics, with the mention of the relative number small number of patients included. The authors emphasize, additionally, the discordance between the gut-saliva bacterial diversity among patients with SLE (an aspect that can be attributed in part to the lack of damage to the oral mucosa of the subjects) and the connection between certain bacterial species (*Lactobacillus*, *Bifidobacterium*, *Akkermansia faecal* and *Ruminococcus*) and the degree of activity of the disease or the modulation of the constituents of the immune response by being involved in maintaining the homeostasis of the intestinal barriers and optimizing regulatory T cells [86]. The link between intestinal dysbiosis and disease activity was also emphasized by Vieira JRP. et al. and Silverman GJ. et al. [84,91].

Regarding the clinical picture of subjects with SLE, Visitación N. et al. postulate, through the study of changes in the cardiovascular system in relation to the intestinal microbiota in murine models, the link between this and the development of endothelial dysfunction, vascular inflammation and hypertension [92]. Lupus nephritis, one of the frequent and feared complications of SLE, seems to have part of its origin in the alteration of intestinal permeability (in addition to aspects such as genetic factors and the individual characteristics of the subject), an aspect that leads over time to the potentiation of autoimmune responses due to metabolites of bacteria that mimic autoantigens, leading to chronic inflammation mediated by lymphocytes and macrophages, activation of the complement and its deposition at the renal level, with intestinal dysbiosis thus becoming one of the possible targets in nephropathy therapy [93,94].

Another direction worthy of consideration is represented by the effects of the anti-rheumatic medication used in the treatment of SLE on the intestinal microbiota, but also by the impact of vitamin D deficiency on the composition of the microbiome, the viability of the intestinal epithelial barriers and autoimmunity (both indirectly and through the effect on regulatory T cells, T helper 1 and T helper 17 differentiation, B cell development and function and monocyte activity) [82,95]. Also, current research aims at the involvement of gender characteristics, such as the influence of sex hormones (estrogen and testosterone), in the intestinal microenvironment, thereby imprinting the biological picture of SLE, but also the ability of the *Ruminococcus gnavus* strain from subjects with lupus to produce alteration of intestinal permeability through the action on zonulin, at the same time documenting the reversal of the process upon initiation of treatment with larazotide acetate (zonulin antagonist) [96,97].

## 7. Modulation of Intestinal Microbiota in the Adjuvant Therapy of SLE

In addition to the standard therapy used in SLE and adapted to pediatric criteria, which includes glucocorticoids, antimalarials, nonsteroidal anti-inflammatory substances, immunosuppressants and biological agents directed against B cells, modern medicine also brings to the fore new approaches such as the impact played by dietary principles and supplementation with probiotics/prebiotics or symbiotics, but also the role of fecal matter transplantation from a healthy donor in the regulation of intestinal homeostasis or future directions that still require certification through human studies [98,99].

The basic characteristic necessary for any microenvironment to survive is its ability to reach the level of symbiosis that can confer some resistance to colonization due in part to the efficient use of nutrients, thus impoverishing the resources needed by pathogens [42]. Occupying a central place in the nutritional pyramid, the appropriate diet seems to imprint the symptomatic course of patients with SLE through its effect on the integrity of the intestinal microbiota. Although the means of action are still incompletely known, the effects of a low fiber dietary intake (characteristic of the western region) among patients with SLE have been shown to be the disruption of autoimmunity, with the escalation of antibody production and the worsening of mortality, while obesity, correlated contrary to the amount of fiber present in the diet, seems to act synergistically at the body level promoting chronic inflammation, intestinal changes and autoimmunity. Another important constituent is represented by water, more precisely its pH, which has proven its effect on the *Firmicutes*/*Bacteroidetes* ratio, just as vitamin A promotes the development of *lactobacilli*, and starch interacts with pathogens, improving mortality and de-escalating inflammation [86,100]. Considering the previous data, we emphasize the importance of oral supplementation with retinoic acid (a metabolite of vitamin A) and vitamin D, as well as reducing salt intake, which, in addition to improving blood pressure, promotes the growth of metabolic products with an anti-inflammatory role (similar results being observed in the transplantation of fecal matter taken from healthy donors) [101]. Wang X. et al. reiterate the benefits of using *Lactobacillus* in the probiotic treatment of SLE and also underline the strong correlation between it and the dietary components [102].

Mu Q. et al. have demonstrated, in murine models, the benefit brought by supplementation with *Lactobacillus* strains on renal function and mortality reduction in lupus nephritis, but also in the reversal of the IL-10/IL-6 ratio, which is a marker of inflammation, the results being objective in a discordant, gender-dependent manner [103]. Similar evidence was objectified in the case of *Bacteroides fragilis*, where the range of action also includes effects on the expression of CD1d, CD86 and the balance between helper T lymphocytes 17 and regulatory T lymphocytes [104]. Administration of probiotics can also have a beneficial effect on cardiovascular function (affected in SLE) by influencing the T helper 17-lymphocyte triad-endotoxinemia-autoantibody production, the hypothesis issued by Visitación N. et al. but this could not be confirmed/disconfirmed in part due to the absence of studies that include large groups of patients diagnosed with SLE who present arterial hypertension as a comorbidity [105].

Thus, summarizing the specialized literature of Esmaeili SA. et al. concludes that the main bacteria intended to restore immunological tolerance by acting on the pro-inflammatory–anti-inflammatory balance, competitive exclusion and antibacterial action are strains of *Lactobacillus* and *Bifidobacteria* [106]. In a similar way, by using the randomized, double-blind study, Widhani A. et al. reveal an improvement in the SLE activity score observed after the administration of a symbiotic preparation based on *Lactobacillus helveticus*, *Bifidobacterium infantis* and *Bifidobacterium bifidum*, for 60 days, which is a finding doubled by the increase in the *Firmicutes*/*Bacteroidetes* ratio (insignificant difference compared to placebo) and the reduction of a number of 13 operational taxonomic units incriminated as possibly pathogenic [107].

Transplantation of large feces, introduced into clinical practice for the purpose of treating *Clostridium difficile* infection, is gaining more and more interest regarding its use in the management of autoimmune diseases (rheumatoid/psoriatic arthritis, multiple sclerosis, type 1 diabetes, SLE, celiac disease, Hashimoto’s thyroiditis, Grave’s disease or Sjogren’s syndrome) and intestinal dysbiosis. Although still lagging behind in terms of research, benefiting so far from little interest, an aspect highlighted by the limited presence of human studies in contrast to those on animal models, it is important to know the advantages of its use, but also the interactions with pharmacological substances, such as antibiotics or laxatives, which can disrupt success rates [74,108,109,110]. However, Huang C. et al. report, in a study carried out on 24 patients in the active phase of SLE, the change in intestinal composition post-administration, without objectifying any serious adverse reactions [111]. Modern directions regarding adjuvant therapy in SLE include oral antibiotic therapy, development of vaccines against disruptive pathogens (a hypothesis confirmed in murine models), modulation of intestinal autophagy and miRNA therapy, as well as the use of mesenchymal stem cells proven to be effective in regulating homeostasis disturbed in intestinal inflammatory diseases [101,112].

## 8. Conclusions

In conclusion, both the study of SLE and that of the internal microenvironment represent two vast research subjects, both found at the pediatric age. The challenge in this stage is represented by the escalation of the pathogenic process of lupus, objectified by more aggressive clinical manifestations and increased mortality in contrast to the adult population. At the other pole of interest, we find the microbiome, a structure of a complexity comparable to that of the human genome, which is in continuous development starting from the intrauterine period until the age of senescence. Numerous studies in the recent literature link the microbial balance to the pathogenesis of SLE, an aspect intensively researched but still incompletely certified. Therefore, given the fragility and increased variability of the microbiota that can be modulated by various exogenous or endogenous factors, but also the connection between it and SLE, we consider it necessary for the clinician to acquire an “overall view” in order to reduce the burden induced by the disease in especially on the child, whose existence is under the sign of continuous discovery and change that must, as much as possible, take place in a natural course. The present work is considered by the authors to be a point of intersection of research on the stated themes, being centered on the succinct description of the main defining aspects regarding the two entities, diagnostic criteria and principles of locating and optimal collection of evidence, and the way in which they can influence each other and the potential impact of adjunctive therapeutic means to pharmacological ones in the evolutionary course of SLE, an aspect that will open new horizons in future research, also emphasizing the vast involvement of microbial disturbances in multiple systemic diseases.

## Figures and Tables

**Table 1:** Diagnostic lines in SLE and dysbiosis (adapted from Fava, A. et al., Levy, D.M. et al., Tucker, L.B. et al. and Pistone, D. et al.) [18,23,24,25].

Systemic Lupus Erythematosus		Dysbiosis
		Sampling methods
Clinical exam	– Fever, fatigue, lymphadenopathy, downward weight curve; – Acute, subacute or chronic skin damage (photosensitive); – Oral/nasal ulceration; – Alopecia; – Vasculitis; – Livedo reticularis; – Subungual telangiectasia; – Raynaud’s phenomenon; – Synovitis, serositis (Pericarditis, pleuritis), symmetrical polyarthritis at the metacarpophalangeal, proximal interphalangeal and knee joints (rarely erosive); – Neuropsychiatric manifestations (convulsions, psychosis, mononeuritis multiplex, myelitis, peripheral or cranial neuropathy and acute confusional state); – Renal damage objectified by proteinuria, cellular casts and alteration of renal function;	pre-moisten swabs skin surface scrapes tape strips skin biopsies nasal tamponade; nasal wash; nasopharyngeal mucus examination; saliva/sputum; oral tamponade; hypopharyngeal/bronchoalveolar/gastric aspirate; pharyngeal exudate; bronhoalveolar lavage; brushing/bronchial biopsy; stool sample; rectal tamponade; urine/semen/vaginal secretions examination; vaginal scraping/biopsy;
Clinical investigations	– Blood count: pancytopenia (leukopenia/lymphopenia, thrombocytopenia, hemolytic anemia); – Renal tests (urea, creatinine); – Immunological investigations (ANA, anti-dsDNA, anti-Smith, anti-phospholipid, anti-ribonuclear, anti-Ro, anti-La, hypocomplementemia, and direct coombs test); – Skin/renal biopsy; – Ultrasonography; – Spinal puncture with cerebrospinal fluid analysis; – MRI;	
Diagnosis	ACR (1997) Skin eruptions of the malar/discoid type; Objectives of photosensitivity; Oral or nasal ulcers; Non-erosive arthritis at the level of the second/several joints; Serositis; Renal manifestations; Neurological manifestations; Hematological manifestations; Immunological abnormalities; SLICC – Lupus nephritis + one clinical and one immunological criterion; EULAR/ACR	
Remarks		
Differential diagnosis in childhood: ✓ Cytomegalovirus, Epstein Barr, Parvovirus 19, HIV; ✓ Bacterial sepsis, Brucella, Leptospira; ✓ Q fever, tuberculosis, Lyme disease; ✓ Leukemia, lymphoma, neuroblastoma, histiocytosis; ✓ Autoimmune diseases; ✓ Medications that induce lupus;		

SLICC—Systemic Lupus Collaborating Clinics, EULAR—European League of Associations for Rheumatology, ACR—American College of Rheumatology, ANA—anti-nuclear antibodies, anti-ds-DNA—antibodies against double-stranded DNA.

**Table 2:** The connection between the disturbance of the main microbial sites and organic diseases.

Microbial Site	Affections Found in Dysbiosis
Skin	– atopic/seborrheic dermatitis; – acute urticaria; – acne; – psoriasis; – skin malignancies;
Respiratory system piratory system	– acute otitis media; – chronic rhinosinusitis; – bronchiolitis; – pneumonia; – asthma; – post damage scaffolds;
Genitourinary system	– bacterial vaginitis; – pelvic inflammatory diseases; – hysteromyoma; – endometriosis/adenomyosis; – sexually transmitted infections; – infection with papilloma virus/cervical dysplasia; – neonatal infections; – spontaneous abortion; – premature birth; – affecting fertility; – oncological pathology of the prostate; – kidney stones; – urinary tract infections;
Gastrointestinal system	– dental caries; – inflammatory bowel diseases; – celiac disease; – diabetes; – autism; – Henoch-Schonlein purpura; – Wiskott-Aldrich syndrome; – appendicitis; – sleep apnea syndrome; – chronic gastritis; – duodenal ulcer; – osteoarthritis; – psoriasis; – acne vulgaris; – atherosclerosis/thrombosis; – obesity; – hyperlipidemias; – depression; – Alzheimer’s/Parkinson’s disease; – schizophrenia; – multiple sclerosis; – neoplasms (oral cavity, esophagus, stomach, lung, pancreas, colorectal);

## Data Availability

No new data was generated.
